# Risk Factors for Progression of Age-Related Macular Degeneration: Population-Based Amish Eye Study

**DOI:** 10.3390/jcm11175110

**Published:** 2022-08-30

**Authors:** Muneeswar G. Nittala, Federico Corvi, Jyotsna Maram, Swetha B. Velaga, Jonathan Haines, Margaret A. Pericak-Vance, Dwight Stambolian, SriniVas R. Sadda

**Affiliations:** 1 Doheny Image Reading Center, Doheny Eye Institute, Los Angeles, CA 90033, USA; 2 Department of Epidemiology & Biostatistics, Case Western Reserve University, Cleveland, OH 44106, USA; 3 John P. Hussman Institute for Human Genomics, University of Miami Miller School of Medicine, Miami, FL 33136, USA; 4 Department of Ophthalmology, Perelman School of Medicine, University of Pennsylvania, Philadelphia, PA 19104, USA; 5 Department of Ophthalmology, David Geffen School of Medicine at UCLA, Los Angeles, CA 90095, USA

**Keywords:** age-related macular degeneration, Amish eye study, complete retinal pigment epithelial and outer retina atrophy, geographic atrophy, optical coherence tomography

## Abstract

Objective: To evaluate the optical coherence tomography (OCT)-based risk factors for progression to late age-related macular degeneration (AMD) in a population-based study of elderly Amish. Methods: A total of 1332 eyes of 666 consecutive subjects who completed a 2-year follow-up visit were included in this multicenter, prospective, longitudinal, observational study. Imaging features were correlated with 2-year incidence of late AMD development. Odds ratios for imaging features were estimated from logistic regression. Baseline OCT images were reviewed for the presence of drusen volume ≥0.03 mm^3^ in the central 3 mm ring, intraretinal hyperreflective foci (IHRF), hyporeflective drusen cores (hDC), subretinal drusenoid deposits (SDD), and drusenoid pigment epithelium detachment (PED). Subfoveal choroidal thickness, drusen area, and drusen volume within 3 and 5 mm circles centered on the fovea were also assessed. Results: Twenty-one (1.5%) of 1332 eyes progressed to late AMD by 2 years. The mean age of the study subjects was 65 ± 10.17 (±SD) years and 410 subjects were female. Univariate logistic regression showed that drusen area and volume in both 3 mm and 5 mm circles, subfoveal choroidal thickness, drusen volume ≥ 0.03 mm^3^ in the 3 mm ring, SDD, IHRF, and hDC were all associated with an increased risk for development of late AMD. The multivariate regression model identified that drusen volume in the 3 mm ring (OR: 2.59, *p* = 0.049) and presence of IHRF (OR: 57.06, *p* < 0.001) remained as independent and significant risk factors for progression to late AMD. Conclusions: This population-based study confirms previous findings from clinic-based studies that high central drusen volume and IHRF are associated with an increased risk of progression to late AMD. These findings may be of value in risk-stratifying patients in clinical practice or identifying subjects for early intervention clinical trials.

## 1. Introduction

Age-related macular degeneration (AMD) affects almost 200 million people worldwide in 2020, which will increase to 288 million by 2040 [1]. AMD is a complex disease in which demographic and environmental factors—including age, gender, smoking, and diet—contribute to the risk of developing disease [2,3]. Despite the complexity of the phenotype, eyes with AMD are classified in different stages of severity in which late stages are defined by the presence of macular neovascularization (MNV) and/or geographic atrophy (GA).

Considering the GA progression, several trials are ongoing to test different agents to determine whether they can slow the progression of the atrophic changes. However, progression of AMD is not fully understood and is highly variable [4]. Increasingly, there has been a desire to intervene at an earlier stage in these AMD patients before irreversible atrophic lesions appear [5]. In order to design such studies, however, it essential to identify biomarkers and patients who are at greatest risk for progression to late AMD.

Recently, OCT has been introduced as an essential tool for identifying atrophy. The Classification of Atrophy Meetings (CAM) group defined the terms complete retinal pigment epithelial (RPE) and outer retinal atrophy (cRORA) using specific OCT criteria [6].

In addition, a number of OCT risk factors for progression to atrophy have been defined in retinal clinic studies and post-hoc analysis of retina clinical trials. These risk factors include central drusen volume ≥0.03 mm^3^, intraretinal hyperreflective foci (IHRF), hyporeflective drusen cores (hDC), and subretinal drusenoid deposits (SDD) [7,8,9,10,11,12]. A retina clinic population, however, may be confounded by selection bias and may not reflect the diversity of AMD in the general population.

The Amish migrated from eastern Europe to the U.S. in the early 18th century to escape religious persecution. Due to the founder effect, genetic changes within the Amish population have increased in frequency making it possible to identify genetic anomalies with a much smaller sample size than would be needed from the general population. Therefore, genetic studies on the Amish afford researchers the opportunity to identify variants of European origin without the burden of recruiting a much larger number of subjects. As the Amish population is a founder population which is genetically and culturally isolated, it features a uniform environment and reduced genetic diversity. Moreover, the Amish do not indulge in smoking, reducing the amount of AMD risk attributable to smoking. The possibility to study this population represents a unique opportunity to evaluate in a clear view the impact of different risk factors in the progression of late AMD. Thus, the purpose of this study is to evaluate the suggested OCT risk factors for atrophy in the context of a longitudinal prospective study from the patients enrolled in the AMISH eye study.

## 2. Methods

The Amish eye study is a multicenter, prospective, longitudinal, observational study with NEI-supported clinical research investigation aimed at understanding genetic associations with AMD progression. Of the 1339 subjects (2668 eyes) who were initially enrolled in the Amish eye study, the first 666 consecutive subjects (49.7%; 1332 eyes or roughly half the cohort) returned for their 2-year follow up visit and were thus included in this analysis. Patients were identified from three settlements in Ohio, Indiana, and Pennsylvania. The human subject protocols have been approved by the Institutional Review Boards of the University of Pennsylvania, University of Miami, Case Western Reserve University, and University of California Los Angeles. All study procedures followed the tenets of the Declaration of Helsinki. Written informed consent was obtained from all subjects after explaining the nature and possible consequences of the study. Inclusion criteria were: (1) age ≥ 50 years; (2) self-identification as Amish; (3) membership in a sibship with at least 1 individual reported to have AMD; (4) follow-up data for at least 2 years. Exclusion criteria included inadequate image quality, any previous or concomitant ophthalmological condition that could confound the interpretation of AMD features on imaging, and presence of late AMD in the study eye at baseline.

### 2.1. Study Design

The data collection and imaging protocols were detailed in previous Amish eye study reports [13]. Data collection was standardized and included measurements taken by clinical research coordinators, ophthalmic technicians, and board-certified ophthalmologists in respective study sites. Patients were evaluated after a 2-year follow-up to assess which cases progressed and which cases did not progress to late AMD.

### 2.2. Study Procedures

Demographic information collected at baseline and at the 2-year follow-up visit included age, sex, family history of AMD, and the presence of any other eye diseases. All individuals received a comprehensive eye exam, including visual acuity measured using a Snellen chart at 20 feet, and a panel of multimodal imaging studies consisting of stereo color fundus photographs (CFPs), blue light fundus autofluorescence (FAF; Spectralis), infrared reflectance (IR; Spectralis), and dense-volume OCT (Cirrus and Spectralis).

#### 2.2.1. OCT Image Acquisition

All structural OCT images were acquired by both the Cirrus HD-OCT (Carl Zeiss Meditec, Dublin, CA, USA), using a macular cube protocol of 128 horizontal B-scans and 512 A-scans per line (6 × 6 mm, foveal centered), and the Spectralis OCT (Spectralis; Heidelberg Engineering, Heidelberg, Germany), using a macular cube protocol of 97 horizontal B-scans and 512 A-scans per line and automatic real-time tracking (ART) of 9 (6 × 6 mm, foveal centered and EDI mode off). In addition to the fovea-centered scan, an additional OCT volume was captured centered on the optic disk. Two devices were originally incorporated in the study to take advantage of the unique capabilities of each instrument, such as the automated drusen quantification by the Cirrus and the better visualization of the outer retinal substructures and choroid by the averaged scans of the Spectralis. Scans were repeated in the case of motion artifacts and/or low signal strength (SS). In accordance with the manufacturer’s recommendations and the reading center’s guidelines, to be acceptable for inclusion, an OCT volume had to have SS > 30 on Spectralis and SS ≥ 7 on the Cirrus.

#### 2.2.2. Structural OCT Image Analysis

**Figure 1:**
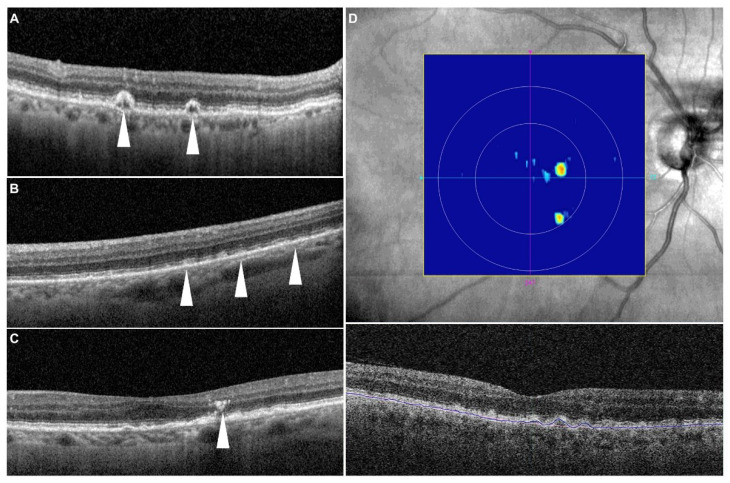
Representative examples of structural optical coherence tomography risk factors. (**A**) Drusenoid lesions with a hyporeflective core (white arrowheads)—note there is no evidence of any shadow artifact to explain the hyporeflectivity; (**B**) subretinal drusenoid deposits (white arrowheads); (**C**) intraretinal hyperreflective focus (white arrowhead)—here seen above the apex of the drusen; (**D**) drusen volume map from the Cirrus Advanced RPE analysis software—the algorithm segments the RPE band following the RPE contour and also estimates the original RPE position (termed the RPE fit) in the absence of an RPE elevation. The region between the RPE band and the RPE fit is then quantified as the drusen volume.

All images were reviewed by two independent and masked graders (MGN and SBV) at baseline and after 2 years for the presence of cRORA, drusenoid pigment epithelium detachment (PED), IHRF, hDC, SDD, and drusen volume ≥0.03 mm^3^ within 3 mm circle (Figure 1), as well as to measure drusen volume and area as continuous metrics within the central 3 mm and central 5 mm circles and subfoveal choroidal thickness.

A cRORA lesion was identified according to the CAM criteria as containing a region of hypertransmission of at least 250 µm in diameter, a zone of attenuation or disruption of the RPE of at least 250 µm in diameter, evidence of overlying photoreceptor degeneration, and the absence of scrolled RPE or other signs of an RPE tear [6].

Drusen volume and drusen area were analyzed using the manufacturer’s FDA-cleared Cirrus Advanced RPE Analysis software (version 6.0) as previously described [14]. The accuracy and reproducibility of automated drusen segmentation in eyes with non-neovascular age-related macular degeneration has been reported in previous publications [15].

IHRF were identified as well-circumscribed hyperreflective lesions within the neurosensory retina, with a minimum size of 3 pixels and a reflectivity equal to or more than that of the RPE band [16]. hDC lesions were identified based on the presence of hyporeflective spaces within drusen with a minimum height of at least 40 μm as previously reported [17]. SDD were identified as medium-reflective to hyperreflective mounds or cones, either at the level of the ellipsoid zone or between the ellipsoid zone and the RPE surface [9]. Subfoveal choroidal thickness was measured using the caliper tool on the central B-scan in the Spectralis device. Calipers were set at the Bruch’s membrane line and the inner surface of the sclera to obtain thickness measurements.

### 2.3. Statistical Analysis

**Figure 2:**
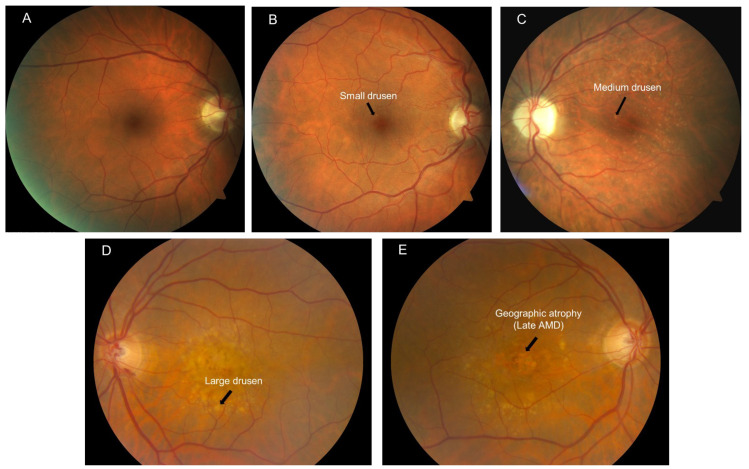
Illustration of the Beckman’s clinical classification of AMD based on color fundus photos. (**A**) No apparent aging changes; (**B**) Normal aging changes; (**C**) Early AMD; (**D**) Intermediate AMD; (**E**) Late AMD evidenced by geographic atrophy.

The primary objective of the study was to evaluate structural OCT biomarkers for their ability to predict progression from intermediate AMD to late AMD, defined as the appearance of cRORA (based on Beckman AMD classification scale, Figure 2). Data analyses were conducted with IBM SPSS (version 26.0; IBM Corporation, Armonk, NY, USA). The normality of sample distribution was evaluated by Kolmogorov–Smirnov and Shapiro–Wilk tests. The χ^2^ test and Student’s t-test for independent samples were used to evaluate differences in proportions and means among categorical and continuous variables, respectively. Logistic regression (univariate followed by multivariate) was also performed, using baseline drusen area in the 3 mm and 5 mm circles, drusen volume ≥0.03 mm^3^ in the 3 mm and 5 mm rings, drusenoid PED, SDD, subfoveal choroidal thickness, IHRF, and hDC as independent variables and the progression to cRORA as the dependent variable. Intraclass correlation coefficient (ICCs) and Cohen’s kappa (κ) were computed to measure the intergrader repeatability for quantitative and qualitative assessments. A *p* value < 0.05 was considered to be statistically significant.

## 3. Results

A total of 1332 eyes of 666 consecutive subjects with a two-year follow-up were included in this analysis. Of these 1332 eyes, twenty-one eyes (1.58%) progressed to late AMD at 2 years.

The mean age of the cohort in this analysis was 65.43 ± 10.17 years and 410 were females (61.56%). Patients who progressed to cRORA within 2 years were older (76.30 ± 6.01 years compared to 65.19 ± 10.14 years for those who did not progress; *p* < 0.001) and more likely to be male (52.9% male among those progressing to cRORA vs. 38.1% among those who did not progress; *p* < 0.001).

**Figure 3:**
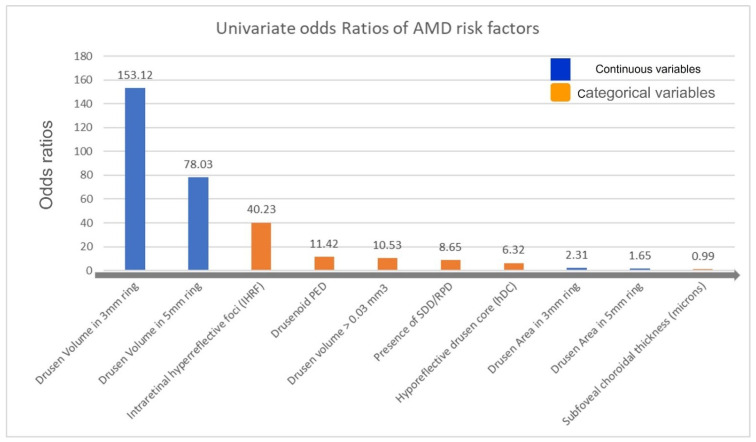
Bar plot showing the odds ratios from the univariate regression analysis (gray arrow indicates the direction of higher to lower risk for late AMD development).

The univariate logistic regression comparing eyes which progressed to cRORA and those which did not progress demonstrated that drusen area in the 3 mm and 5 mm ring, drusen volume ≥0.03 mm^3^ in the 3 mm and 5 mm ring, subfoveal choroidal thickness, SDD, IHRF, and hDC were all associated with an increased probability of cRORA development (OR = 2.31, 95% CI 1.65–3.23, *p* < 0.001), (OR = 1.65 95% CI 1.33–2.05, *p* < 0.001), (OR = 153.12, 95% CI 8.98–2610; *p* = 0.001), (OR = 78.03, 95% CI 7.49–813, *p* < 0.001) (OR = 1.01, 95% CI 1.00–1.01, *p* = 0.002), (OR = 8.65, 95% CI 3.49–21.46, *p* < 0.001) (OR = 40.23, 95% CI 14.41–112.37, *p* < 0.001), (OR = 4.1, 95% CI 2.77–6.07, *p* < 0.001) (Table 1 and Figure 3). The multivariate regression model confirmed the association between drusen volume ≥0.03 mm^3^ in the 3 mm ring and IHRF (Table 1).

Following a multivariate regression analysis performed by removing collinear variables like drusen area and drusen volume in 5 mm circles, results showed IHRF (OR: 37.66, (95% CI 10.75–131.98, *p* < 0.001) is still an independent and significant risk factor for late AMD progression.

## 4. Discussion

In this study, we evaluated structural OCT biomarkers for their ability to predict progression from intermediate AMD to cRORA in the prospective Amish eye study. The univariate analysis revealed that previously identified OCT risk factors appeared to predict progression to late AMD in this cohort. Importantly, IHRF and drusen volume in the 3 mm circle remained independent predictors of progression even when other structural OCT biomarkers were considered.

A few studies have evaluated the relationship between specific AMD biomarkers and AMD progression. Recently, Lei et al., proposed an OCT-based scoring system for the progression of AMD which included the presence of IHRF, SDD, hDC, and drusen volume within a central 3 mm circle ≥0.03 mm^3^ [12].

Consistently, across multiple studies, IHRF have been identified as one of the strongest risk factors for atrophy and late AMD [7,16,18,19,20]. Histopathologic, laboratory, and OCT studies have suggested that IHRF represent the migration of activated RPE cells into the inner retinal layers which may be the result of an inflammatory response due to the oxidative damage and complement-activation processes in the pathophysiology of AMD [16]. Christenbury and colleagues observed that an increasing number of IHRF was associated with a greater incidence of geographic atrophy at 2 years [19]. Nassisi et al., observed a correlation between the quantity of IHRF in the eyes with intermediate AMD and the 1-year risk of progression to late AMD [21]. Recently, the presence of IHRF was also highlighted as an important risk factor by Schmidt-Erfurth et al., using an artificial intelligence model [18]. In our study, IHRF was confirmed to be a strong and independent predictor of late AMD.

Drusen volume ≥0.03 mm^3^ within the central 3 mm of the macula has also been noted be an important risk factor for progression. Abdelfattah and colleagues were able to demonstrate that a drusen volume ≥ 0.03 mm^3^ was associated with a higher risk of progression to late-stage AMD, and in particular to GA [10]. In this study, we confirmed the importance of drusen volume as both a categorical and a quantitative and continuous parameter.

Numerous studies have identified hDC as risk factors for progression to atrophy in several studies [7,12,18,20]. In particular, it was hypothesized that increasing heterogeneity within the drusen (which initially have a homogenous medium-reflective appearance) may reflect progressive impairment of RPE function and impending collapse of the drusen leading to atrophy. Recently, Tan and colleagues were able to show that these hyporeflective cores corresponded histologically to multi-lobular calcific hydroxyapatite nodules that seemed to gradually lead to the complete loss of overlying RPE [22].

SDD are considered another important risk factor in the progression of AMD [9]. In particular, the presence of SDD strongly correlate with the progression from intermediate AMD to late AMD. Marsiglia and colleagues evaluated GA progression in eyes with dry AMD, finding a high correlation between the presence of SDD and the presence and expansion of GA [23]. It has been observed that SDD eyes are generally characterized by a progressive reduction in choroidal and choriocapillaris blood flow during the progression of AMD [24,25]. Interestingly, in this study, SDD and a thin choroid were found to be associated with development of late AMD. In fact, eyes with GA and SDD generally manifest a thinner choroid compared to normal eyes of similar age [26].

Our univariate analysis confirmed that hDC and SDD were significant risk factors for progression from iAMD to cRORA. However, they did not remain significant in the multivariate model when including an IHRF and a high central drusen volume. It is possible that with a longer time frame and larger sample they may have remained significant.

Our study is not without limitations which must be considered when assessing our findings. First, the follow-up evaluation occurred at 2 years which may be a short time in the context of AMD. As a result, there were relatively few progression events in this cohort. Other risk factors may have been identified with a longer follow-up. Second, we used only structural OCT to identify both risk factors and progression to the late AMD end point. Other imaging modalities such as fundus autofluorescence, fluorescein angiography, and OCT angiography were not included in this analysis. On the other hand, structural OCT is widely available, and OCT-based biomarkers may be of the greatest clinical value. Despite these limitations, our study does have several strengths including the use of two masked, independent, experienced reading-center graders for each image and the use of standardized protocols and data collection in the context of a large prospective observational study.

In summary, we observed that the presence of IHRF and a high drusen volume in the central 3 mm ring were independently and significantly associated with development of late AMD. Other previously described AMD biomarkers such as hyporeflective drusen cores and subretinal drusenoid deposits did not remain as independent predictors in the multivariable model, highlighting their collinearity with other factors. This population-based study confirms the findings from previous clinic-based studies regarding the importance of IHRF and high central drusen volume as risk factors for progression to late AMD. These findings may of value in risk-stratifying patients in clinical practice, establishing follow-up intervals for re-assessment, and for identifying subjects for early-intervention clinical trials.

## Figures and Tables

**Table 1:** Regression Analysis to Study the Effect of Various Biomarkers on GA incidence.

	Univariate Analysis			Multivariate Analysis		
	Odds Ratio	95% CI	*p*	Odds Ratio	95% CI	*p*
Drusen volume ≥0.03 mm^3^ in the center 3 mm ring *						
No	1			**1**		
Yes	10.53	4.09–27.09	**<0.001**	3.47	0.42–28.34	0.25
Intraretinal hyperreflective foci (IHRF) *						
No	1			1		
Yes	40.23	14.41–112.37	**<0.001**	57.06	10.02–324.87	**<0.001**
Hyporeflective drusen core (hDC) *						
No	1			1		
Yes	6.32	2.53–15.77	**<0.001**	1.94	0.53–7.03	0.32
Presence of subretinal drusenoid deposits (SDD)/Reticular Pseudodrusen (RPD) *						
No	1			1		
Yes	8.65	3.49–21.46	**<0.001**	0.66	0.17–2.67	0.56
Drusenoid PED *						
No	1			1		
Yes	11.42	4.55–28.69	**<0.001**	1.17	0.23–5.91	0.85
Drusen Area in 3 mm ring ^#^	2.31	1.65–3.23	**<0.001**	0.03	0.001–1.12	0.06
Drusen Area in 5 mm ring *^#^*	1.65	1.33–2.05	**<0.001**	24.06	0.89–652.73	0.06
Drusen Volume in 3 mm ring ^#^	153.12	8.98–2610	**0.001**	2.59	1.46–6.54	**0.049**
Drusen Volume in 5 mm ring ^#^	78.03	7.49–813	**<0.001**	1.49	5.76–38.71	0.06
Subfoveal choroidal thickness (microns) ^#^	0.99	0.985–0.997	**0.002**	1	0.99–1.01	0.65

* Qualitative and categorical parameters; ^#^ Quantitative and continuous parameters; PED—Pigment epithelium detachment.

## Data Availability

Not applicable.

## Abbreviations

AMD	Age-related Macular Degeneration
iAMD	intermediate age-related Macular Degeneration
cRORA	Complete Retinal Pigment Epithelial and Outer Retina Atrophy
iRORA	Incomplete Retinal Pigment Epithelial and Outer Retina Atrophy
OCT	Optical Coherence Tomography
IHRF	Intraretinal Hyperreflective Foci
SDD	Subretinal Drusenoid Deposits
hDC	Hyporeflective drusen cores
RPE	Retinal Pigment Epithelium
MNV	Macular Neovascularization
GA	Geographic Atrophy
OR	odds ratio
